# Sodium bicarbonate-based hydration prevents contrast-induced nephropathy: a meta-analysis

**DOI:** 10.1186/1741-7015-7-23

**Published:** 2009-05-13

**Authors:** Pascal Meier, Dennis T Ko, Akira Tamura, Umesh Tamhane, Hitinder S Gurm

**Affiliations:** 1 University of Michigan School of Medicine, Ann Arbor, Michigan, USA; 2 Institute for Clinical Evaluative Sciences, University of Toronto, Ontario, Canada; 3 Internal Medicine 2, Oita University, Yufu, Japan; 4 VA Ann Arbor Healthcare System, Ann Arbor, Michigan, USA

## Abstract

**Background:**

Contrast-induced nephropathy is the leading cause of in-hospital acute renal failure. This side effect of contrast agents leads to increased morbidity, mortality, and health costs. Ensuring adequate hydration prior to contrast exposure is highly effective at preventing this complication, although the optimal hydration strategy to prevent contrast-induced nephropathy still remains an unresolved issue. Former meta-analyses and several recent studies have shown conflicting results regarding the protective effect of sodium bicarbonate. The objective of this study was to assess the effectiveness of normal saline versus sodium bicarbonate for prevention of contrast-induced nephropathy.

**Methods:**

The study searched MEDLINE, EMBASE, Cochrane databases, International Pharmaceutical Abstracts database, ISI Web of Science (until 15 December 2008), and conference proceedings for randomized controlled trials that compared normal saline with sodium bicarbonate-based hydration regimen regarding contrast-induced nephropathy. Random-effects models were used to calculate summary odds ratios.

**Results:**

A total of 17 trials including 2,633 subjects were pooled. Pre-procedural hydration with sodium bicarbonate was associated with a significant decrease in the rate of contrast-induced nephropathy (odds ratios 0.52; 95% confidence interval 0.34–0.80, *P *= 0.003). Number needed to treat to prevent one case of contrast-induced nephropathy was 16 (95% confidence interval 10–34). No significant differences in the rates of post-procedure hemodialysis (*P *= 0.20) or death (*P *= 0.53) was observed.

**Conclusion:**

Sodium bicarbonate-based hydration was found to be superior to normal saline in prevention of contrast-induced nephropathy in this updated meta-analysis.

## Background

Contrast agents are administered to millions of procedures annually worldwide. In the USA and Europe, contrast-induced nephropathy (CIN) is the third leading cause of acute renal failure in hospitalized patients, accounting for about 10% of hospital-acquired renal failure [1]. The issue of contrast agent-related risk further gains importance with the growing awareness that even gadolinium-based agents are not harmless and may induce renal damage [2].

Two mechanisms have been hypothesized to be responsible for CIN development: contrast media-triggered vasoconstriction and development of oxidative stress, that is, intrarenal accumulation of reactive oxygen species. One major underlying hypothesis for application of sodium bicarbonate (NaHCO_3_) is that the alkalinization of tubular fluid diminishes the production of free oxygen radicals [3,4]. Pretreatment with sodium bicarbonate is more protective than sodium chloride in animal models of acute ischemic renal failure [5].

Previous meta-analyses have shown a significant benefit for NaHCO_3 _in comparison to normal saline (NS) infusion [6,7], although they highlighted the possibility of publication bias. Two trials published thereafter did not show an advantage of NaHCO_3 _[8,9], while multiple smaller studies have published preliminary but rather contradictory results [10,11]. A recent retrospective study suggested even possible harm of NaHCO_3 _[12]. Given these conflicting results, an updated meta-analysis is meaningful. The purpose of this study was to evaluate the current published and unpublished data regarding the use of NaHCO_3 _versus NS as pre-procedural hydration for the prevention of CIN.

## Methods

We searched PubMed, MEDLINE, the Cochrane Central Register of Controlled Trials, International Pharmaceutical Abstracts database, and ISI Web of Science and google scholar from 1990 through to 15 December 2008. In addition, abstract lists and conference proceedings from the 2007 and 2008 scientific meetings of the American College of Cardiology, the European Society of Cardiology, the Transcatheter Cardiovascular Therapeutics, the American Heart Association, the American Society of Nephrology, the European Renal Association, the annual meeting of the Radiology Society of North America Annual, and the World Congress of Cardiology were searched. We also considered published review articles, editorials, and internet-based sources of information (, ) to assess for potential information on studies of interest. Medical subject headings and keyword searches included the terms 'contrast nephropathy', 'sodium bicarbonate', 'saline infusion', 'radiocontrast', and 'renal failure'. Reference lists of selected articles were reviewed for other potentially relevant citations. Authors of selected studies were contacted to obtain further information.

### Study selection

**Figure 1:**
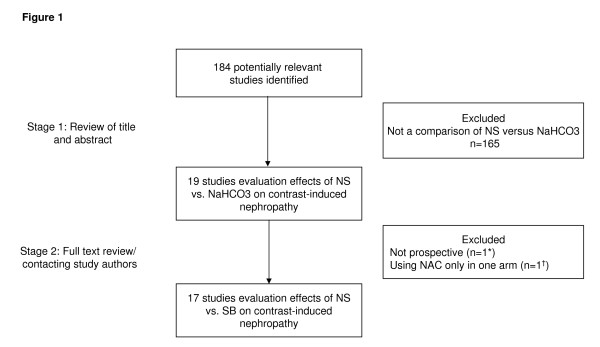
**Flow chart depicting outline of the search and selection strategy**. NS = normal saline; NaHCO_3 _= sodium bicarbonate; NAC = N-acetylcysteine. *The study of Schmidt *et al*. [13] was not prospective. ^†^Shavit *et al*. randomized patients to NS + N-acetylcysteine versus NaHCO_3_.[14]

In a two-step selection process, two investigators (HSG and PM) independently reviewed the titles and abstracts of all citations to identify all potentially relevant studies. In a second step, the corresponding publications were reviewed in full text by the same two investigators to assess if studies met the following inclusion criteria: direct comparison of NaHCO_3 _versus pre-hydration with NS, randomized controlled trial (RCT), and CIN as primary endpoint based on laboratory testing (Figure 1). Reviewers were not blinded to study authors or outcomes. Final inclusion of studies was based on the agreement of both reviewers. Two studies were excluded because they were either retrospective [13] or because they randomized patients to NS + N-acetyl cysteine (NAC) versus NaHCO_3 _only [14].

### Data extraction and quality assessment

Two reviewers (PM and UT) extracted relevant information from the articles including baseline clinical characteristics of the study population, laboratory data, baseline creatinine, amount of NS infusion in the control group (total and before contrast administration), type of contrast, average contrast volume and data on primary (CIN) and secondary outcomes, such as mortality, need for hemodialysis (HD). CIN was defined differently by each study, but most described it as an absolute or relative increase in serum creatinine. Seven studies defined CIN as a rise in serum creatinine by 25% or more within 2 to 5 days of contrast exposure [15-21]. Two studies regarded an absolute increase of creatinine of 0.5 mg/dl (= 44 μmol/liter) as their primary definition of CIN [9,22], whereas six authors used a composite definition of either a 25% relative creatinine increase or an absolute increase of 0.5 mg/dl [8,11,23-26]. One study [27] used change in glomerular filtration rate as the primary endpoint and change in creatinine as a secondary definition for CIN and one abstract [10] did not mention the CIN definition. For each study we used the corresponding predefined primary endpoint. We assessed trial quality by evaluating specific criteria (concealment of allocation during randomization, intention-to-treat analysis, and blinded assessment of outcome measures), but did not use a quality score in regard to the limitations inherent in such an approach [28].

### Data synthesis and analysis

Data from all the selected studies were combined to estimate the pooled odds ratio (OR) of effect sizes for NS compared with NaHCO_3 _using a random-effects model. All analyses were performed on an intention-to-treat basis. Continuity correction was used when an event did not occur in one group [29]. Significant between-study heterogeneity was expected regarding study populations, therefore a random-effects model was used to produce across-study summary OR with 95% confidence intervals (CI). We evaluated the presence of heterogeneity across trials with the Q and I^2 ^statistics, with an I^2 ^value > 50% indicating at least moderate statistical heterogeneity. To assess the effect of individual studies on the summary estimate of effect, we did an influence analysis, in which the pooled estimates were recalculated by omitting one study at a time. We assessed publication bias visually (funnel plot) and by formal tests (rank order correlation [30] and Egger's test of intercept [31]). A Funnel plot depicts the effect estimates from each trial against study sample size or precision. In absence of publication bias, this plot should appear approximately symmetrical. The Funnel plot is based on the fact that large trials can estimate effects more precisely while smaller trials show wider scattering. A formal measure of Funnel plot asymmetry is the Egger test. It is a linear regression of normalized effects (effect divided by its standard error (SE)) against precision (reciprocal SE of the effect). Very small studies have a larger SE and, consecutively, a normalized effect and a precision that is close to zero. Therefore, the intercept of the regression comes close to zero; if it significantly differs from zero, this indicates systematic deviance of the effect of small studies (small study effect and/or bias, for example, due to publication bias).

We also calculated fail-safe *N *(that is, the number of studies required to nullify the significant differences in CIN between the two groups) using Rosenberg's and Orwin's method [32,33]. As there was a suggestion of publication bias, Duval and Tweedie's [34] Trim and Fill method was used to calculate imputed OR for CIN. Exploratory meta-regression based on a mixed-effect model was performed to estimate the extent to which selected covariates that potentially influence risk for CIN could explain the observed heterogeneity of the NaHCO_3 _effect (average age of patients, baseline creatinine, volume of contrast medium used, prevalence of diabetic patients in the study, and amount of NS infusion before contrast administration in the control group). Stratified analysis was performed to assess the effect of NaHCO_3_-based hydration in select groups (patients treated with low osmolar versus iso-osmolar contrast, and those undergoing elective versus emergent procedures) and to assess impact of published versus unpublished studies. All analyses were performed using Comprehensive Meta-Analysis software, version 2.0 (Biostat, Englewood, NJ, USA) and MIX, version 1.7 [35].

## Results

A total of 184 articles were reviewed, and 17 studies satisfied the predetermined inclusion criteria (Figure 1) [8-11,15-27]. Eight trials have been published in the peer-reviewed literature whereas another nine studies are as yet unpublished but were presented at scientific meetings (that is, Transcatheter Therapeutics meeting, American Heart Association meeting, American Society of Nephrology meeting, World Congress of Cardiology, or Canadian Cardiovascular Congress). Further information on these unpublished trials was obtained from the study authors where possible. One study randomized patients to three arms: NaHCO_3_, NS, or NS and NAC. From this trial, we used data only from the NaHCO_3 _and NS arms and excluded the patients randomized to the NS and NAC arm [25]. Data from studies using a 2 × 2 factorial-design testing the influence of NAC and the effect of NaHCO_3 _were used by pooling patients from the NaHCO_3 _and NS arms (with or without NAC) [18,26]. Additional file 1 summarizes the characteristics of the 17 trials, including a total of 2,633 subjects.

### Primary endpoint

**Figure 2:**
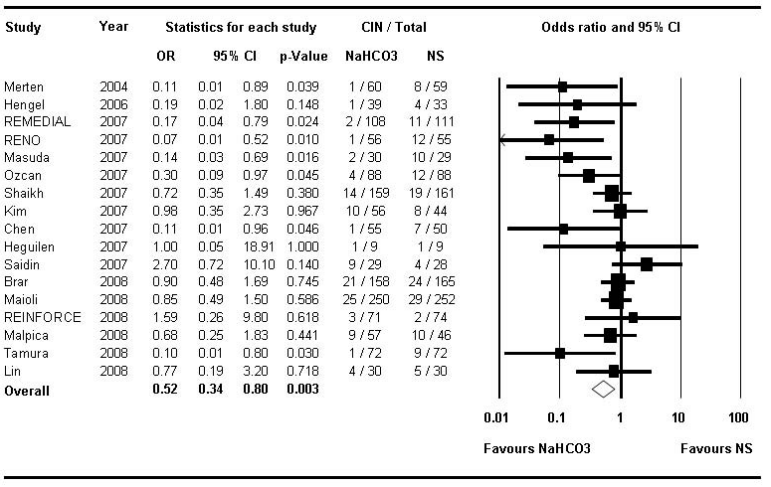
**The Forest plot of odds ratios of contrast-induced nephropathy**. Sizes of data markers are proportional to the weight of each study in the meta-analysis. Studies are stratified by year of presentation and/or publication. Horizontal bars, 95% confidence interval. NaHCO_3 _= sodium bicarbonate; NS = normal saline.

CIN occurred in a total of 109 patients in the 1,327 patients of the NaHCO_3 _arms (range 1.4% to 31.0%) compared with 175 such events in the 1,306 subjects treated with NS (range 2.7% to 34.5%) (Table 1). The summary OR was 0.52 (95% CI 0.34–0.81, *P *< 0.004) (Figure 2) in favor of NaHCO_3_. The number needed to treat to prevent one CIN was 16 (95% CI 10–34).

**Table 1:** Incidence of contrast-induced nephropathy in treatment arms.

Study	CIN incidence NaHCO_3_ (%)	CIN incidence NS (%)
Merten	1.7	13.6
Hengel	2.6	12.1
REMEDIAL	1.9	9.9
RENO	1.8	21.8
Masuda	6.7	34.5
Ozcan	4.5	13.6
Shaikh	8.8	11.8
Kim	17.9	18.2
Chen	1.8	14.0
Heguilen	11.1	11.1
Saidin	31.0	14.3
Brar	13.3	14.5
Maioli	10.0	11.5
REINFORCE	4.2	2.7
Malpica	15.8	21.7
Tamura	1.4	12.5
Lin	13.3	16.7

CIN = contrast-induced nephropathy; NaHCO_3 _= sodium bicarbonate; NS = normal saline.

There was moderate heterogeneity across studies regarding clinical patient characteristics and protocols, and also formal tests (I^2 ^= 48.0; Q = 30.6; *P *value = 0.015).

**Figure 3:**
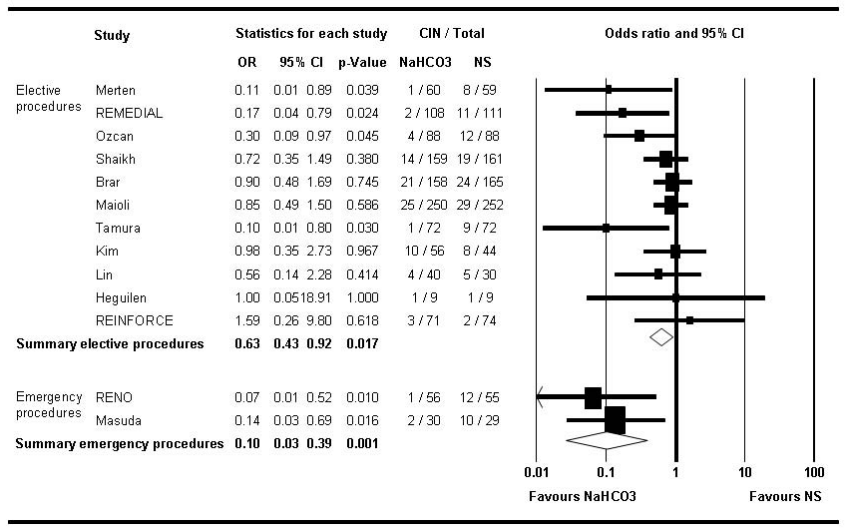
**The Forest plot of odds ratios of contrast-induced nephropathy**. This is stratified by studies with elective procedures versus those including only emergency procedures. Sizes of data markers are proportional to the weight of each study in the meta-analysis. Horizontal bars, 95% confidence interval.

**Figure 4:**
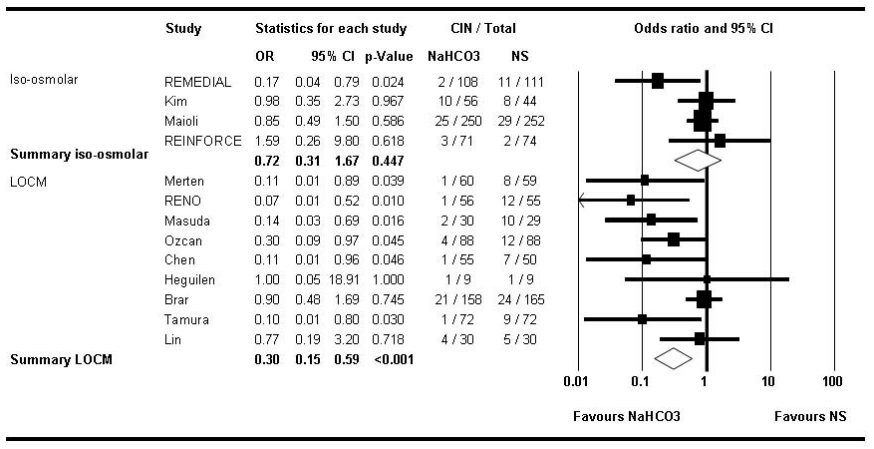
**Forest plot of stratified analysis by studies using iso-osmolar (iodixanol) versus low-osmolar contrast media**. Sizes of data markers are proportional to the weight of each study in the meta-analysis. Horizontal bars, 95% confidence interval.

Stratified analyses suggested a more pronounced effect of NaHCO_3 _in the two trials including exclusively patients undergoing emergency procedures [22,24] (OR 0.10; 95% CI 0.02–0.42; *P *= 0.002) compared with patients undergoing elective procedures (OR 0.63; 95% CI 0.43–0.92; *P *= 0.017) (Figure 3). Similarly, stratified analysis by the type of contrast medium used suggested lower odds of CIN with NaHCO_3 _in studies using low-osmolar contrast media [11,16,19,20,22-25,27] (OR 0.29; 95% CI 0.15–0.57) compared with those using the iso-osmolar agent iodixanol [8,9,15,18] (OR 0.73; 95% CI 0.32–1.64, *P *= 0.441) (Figure 4).

**Figure 5:**
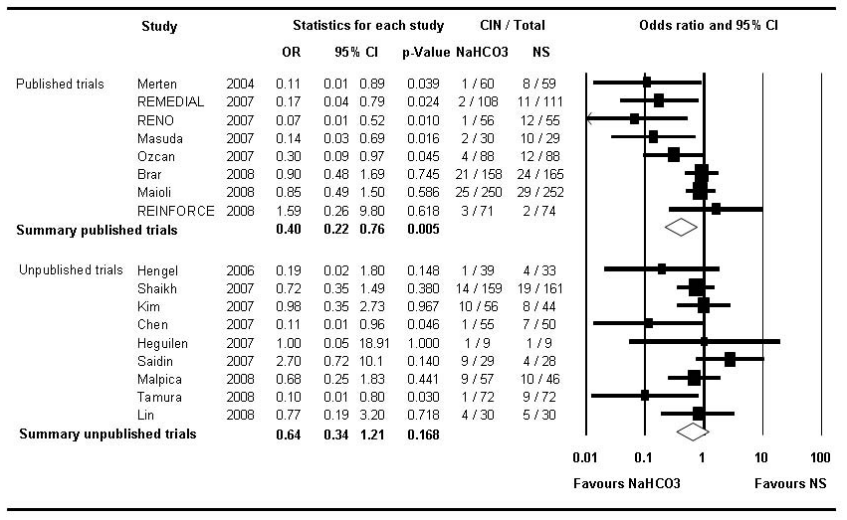
**The Forest plot of odds ratios of contrast-induced nephropathy stratified by publication status**. Sizes of data markers are proportional to the weight of each study in the meta-analysis. Horizontal bars, 95% confidence interval.

Exploratory meta-regression did not identify any significant association between the reduction in CIN with NaHCO_3 _and any of the baseline variables that were tested (baseline creatinine, NAC use, proportion of patients with diabetes, contrast volume). The eight published trials showed a stronger overall benefit of NaHCO_3 _(OR 0.4; 95% CI 0.21–0.76, *P *= 0.005) compared with the nine unpublished studies (OR 0.64; 95% CI 0.34–1.21, *P *= 0.168) (Figure 5). Furthermore within the published trials, those stopped early [20,24], demonstrated a much greater reduction in risk of CIN with NaHCO_3 _(OR 0.12; 95% CI 0.03–0.56, *P *= 0.007) compared with trials that completed enrolment as planned (OR 0.53; 95% CI 0.28–1.0; *P *= 0.051). After exclusion of those two trials, the overall OR based on all other studies was 0.60 (95% CI 0.40–0.91; *P *= 0.016).

**Figure 6:**
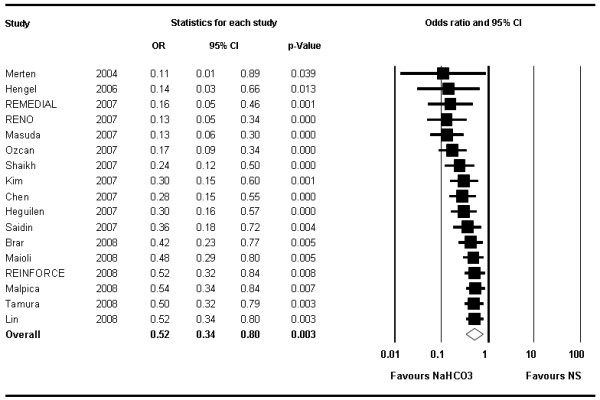
**Cumulative analysis of contrast-induced nephropathy**. This figure depicts the summary odds ratios of all trials published (in the literature or as an abstract) up to a time point in chronologic order. The odds ratios increase over time illustrating that earlier trials found more pronounced effects of sodium bicarbonate than subsequent studies, while the confidence intervals have narrowed suggesting greater reliability of the effect estimate. Horizontal bars, 95% confidence interval.

The cumulative analysis illustrates the time-course of the OR when performing a meta-analysis after each new study in a chronologic order (Figure 6). This suggests that the initial trials were more likely to show dramatic reduction in CIN with NaHCO_3 _but subsequent trials have demonstrated more modest benefits.

**Figure 7:**
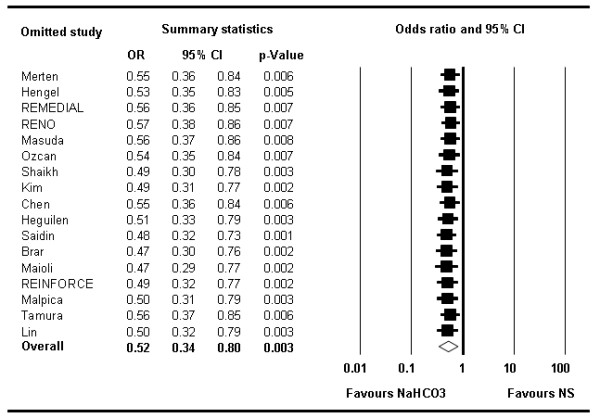
**Forest plot of odds ratios illustrating the influence of single trials on the overall analysis**. Each row represents an overall estimate of odds ratios when omitting one study (left column represents name of omitted study). The lowest row shows the overall odds ratio when all studies are included. No particular study relevantly influences the overall odds ratio. Horizontal bars, 95% confidence interval. NaHCO_3 _= sodium bicarbonate; NS = normal saline.

None of the studies influenced the results to an extent that the conclusion would have changed; the sensitivity analysis omitting one study at a time consistently showed an overall benefit of NaHCO_3 _compared with NS (Figure 7).

**Figure 8:**
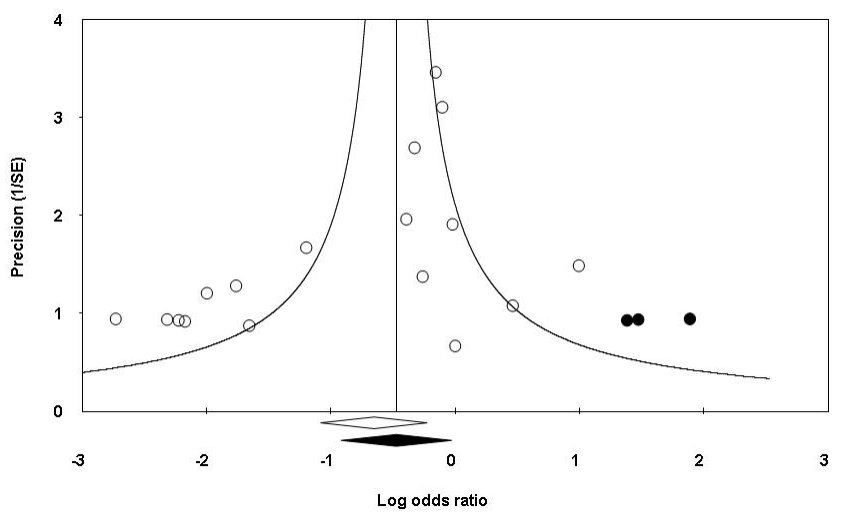
**Funnel plot of contrast-induced nephropathy (with estimated unpublished studies)**. Trials are depicted by circles with the random-effects log odds ratio shown along the horizontal axis and precision in estimating this effect (reciprocal standard error) along the vertical axis. The Trim and Fill method was used to calculate the true center of the funnel (indicated by the vertical line) after filling in estimates of unpublished studies (depicted with black dots). The empty diamond indicates the original confidence intervals of the log odds ratio; the black diamond indicates the corresponding values when the additional imputed studies are also considered.

Assessment of publication bias using a funnel plot indicated slight asymmetry (Figure 8), and this was confirmed on formal testing (rank order correlation or Kendall *τ *of -0.324, one-tailed *P *value of 0.038, two-tailed *P *= 0.077, and Egger's test intercept of -1.71, 95% CI of -3.02 to -0.40 with one-tailed *P *value of 0.007, two-tailed *P *= 0.014). These tests suggest that the results of this meta-analysis were influenced by a larger treatment effect seen in smaller studies.

The Trim and Fill method was used to calculate a treatment effect based on imputed estimated unpublished studies. The imputed OR for CIN using the random-effects model was 0.62 (95% CI 0.40–0.97). The classic fail-safe *N *was 67, suggesting that 67 additional negative studies would be needed to negate the results of our meta-analysis. A much more conservative variant of this method, the Orwin's fail-safe *N*, assumes that unpublished or future studies are not simply negative but rather in disfavor of NaHCO_3_. Under the very conservative assumption of a mean OR of 2.7 in unpublished studies (as observed in the included trial with the most extreme result [21]), eight such trials would be necessary to nullify the overall beneficial effect of NaHCO_3_.

### Secondary endpoints

#### Need for dialysis

The need for HD was reported in 12 studies (*N *= 2,011), in five of these, there was no HD event in either group [8,16,19,20,23]. Overall, 6 out of 934 patients treated with NaHCO_3 _underwent HD (range 0% to 3.3%) compared with 12 out of the 927 patients treated with NS (range 0% to 10.3%). This difference was not statistically significant (OR 0.53; 95% CI 0.20–1.41, *P *= 0.20).

#### Mortality

Data for mortality were available from seven studies (*N *= 1,334). In three studies, no death was observed in either group [11,19,23]. There were a total of eight deaths in the 886 patients treated with NaHCO_3 _(range 0% to 1.8%) and 12 in the 666 patients treated with NS (range 0% to 7.3%). The overall-mortality risk was not significantly different between the two treatment groups (OR 0.74; 95% CI 0.29–1.9, *P *= 0.53).

## Discussion

In this meta-analysis of 17 RCTs including 2,633 patients, pre-procedural hydration with NaHCO_3 _reduced the incidence of CIN compared with hydration with NS. Our findings thus corroborate and extend the prior meta-analysis in the field. Our findings were based on a larger number of trials, include a much larger patient population and adjusted for publication bias by including multiple unpublished studies.

Although CIN is generally limited to a transient decline of renal function, it can not be regarded as a benign complication. Some degree of residual renal impairment has been reported in as many as 30% of those affected by CIN [36]. In an observational study of patients undergoing percutaneous coronary intervention, dialysis was started in 0.8%; persistent renal failure requiring permanent dialysis developed in 13% of these patients [37]; in patients with diabetes and severe renal failure the rate of dialysis was even higher [38]. CIN is associated with a prolonged hospital stay and corresponding additional costs [8,17], and it portends a high morbidity and an increased in-hospital and long-term mortality [39-43]. Acute renal insufficiency leads to increased myocardial oxygen consumption and impaired vascular function in dogs [44] and it is plausible that similar mechanisms may be applicable in humans.

However, this diminished risk for CIN seems not to translate into a decreased incidence of death or need for HD in this current meta-analysis. A similar phenomenon has also been reported in studies evaluating NAC [45]. Several reasons may account for this disconnection between CIN and need for renal replacement therapy or mortality. The patient population enrolled in these studies was generally young and at low risk of adverse events, and very few studies provide long-term follow-up data. The incidence of mortality and need for HD was low in general. Given such low event rates, even a pooled analysis is underpowered to detect a significant difference. Furthermore, it is possible that the definitions of CIN used in these studies are too sensitive regarding the prediction of adverse outcome. Finally, contrast media may impair tubular creatinine excretion, which could lead to an underestimation of the renal function when using creatinine as a surrogate [46]. Importantly, while not significant statistically, the incidence of both death and need for renal replacement therapy was lower in the NaHCO_3 _arm.

While the overall results of the meta-analysis and the imputed OR favors use of NaHCO_3_, possible reasons for heterogeneity must be considered. The study populations and the study settings were rather variable. Stratified analyses revealed differences between NaHCO_3 _effects in emergency and elective cases as one potential reason for the heterogeneity. Interestingly, the benefit of NaHCO_3 _was less prominent in patients treated with iso-osmolar contrast than in those treated with other contrast agents. Recent studies suggest that iodixanol may be less nephrotoxic compared with some (but not all) low molecular weight contrast agents and further studies are warranted to assess the interaction between the type of contrast agent used and the hydration strategy. These *post-hoc *analyses (meta-regressions, subset analyses) must be considered exploratory and hypothesis generating since they are based on a small number of trials and no adjustment for multiple comparisons was made.

The presented overall benefit of NaHCO_3 _may also be slightly overestimated by the two trials that were stopped early (both because of an overwhelming beneficial effect in the treatment group). Early stopping is controversial and it can lead to an overestimation of a treatment effect [47]. However, the early stopped trials are rather small, and their overall influence therefore limited. Even after exclusion of these two studies, the overall benefit of NaHCO_3 _persists.

The importance of publication bias in meta-analysis in general and in this field in particular has been highlighted in the past. The overall magnitude of CIN reduction was clearly greater in published studies compared with unpublished studies. The strength of this study is the inclusion of all available unpublished data and therefore represents the most robust analysis in this field so far. However, even after a comprehensive search for, and inclusion of these unpublished studies, formal testing indicates a likelihood of more unpublished data. Based on statistical estimations, the number and overall effect of unpublished data are probably limited. The treatment effect based on imputed estimated unpublished studies still supports superiority of NaHCO_3 _(OR 0.62; 95% CI 0.40–0.97). The use of NaHCO_3 _is likely associated with a reduction in the incidence of CIN, which may be somewhat overestimated by unpublished studies.

On the other hand, the inclusion of many unpublished studies to overcome the problem of publication bias, introduces the possibility of including lower-quality data that has not undergone robust peer review. However, these trials have all been presented at scientific meetings, published in an abstract format and have undergone a limited peer-review.

While most randomized data suggest either a lower risk of CIN with NaHCO_3 _or no difference compared with NS, a large observational study from the Mayo Clinic has raised the possibility of potential harm with this strategy. In a retrospective study of 7,911 patients encompassing 11,516 cases of contrast exposure, the risk of CIN among patients treated with NaHCO_3 _was significantly increased compared with the untreated group (OR 3.0, *P *< 0.05) [12]. Only a small proportion of patients (489 out of 7,977) were pre-treated with NaHCO_3 _and the extreme results of this study in contrast to the randomized data suggest that the conclusions may be driven by unrecognized confounders. It is reassuring that despite multiple trials performed in various countries, none has demonstrated superiority of saline-based hydration and the overall odds of mortality and need for renal replacement therapy albeit exceedingly low overall, are lower in the NaHCO_3 _arm. The cumulative analysis further supports this conclusion, at no particular time point since the first publication of Merten *et al*. [20], the OR pointed towards an inferiority of NaHCO_3_. However, this also illustrates the fact that the effect of NaHCO_3 _probably was significantly overestimated at first and the inclusion of the recent studies seems to be leading us closer to a more accurate estimate of effect size.

It remains unclear whether there is a dose effect and whether alkalinization is the underlying beneficial mechanism. Six studies monitored the degree of alkalinization (pH urine or blood) [11,15,19,20,22,24]. All but one found a significant increase in pH with exception of Lin *et al*. [19], which in fact was the only one among them not to find a benefit of NaHCO_3_. Therefore, it could be hypothesized that NaHCO_3 _should be dosed to achieve urinary alkalinization. Promisingly, the study of Tamura *et al*. [11] indicates that even a single bolus of NaHCO_3 _administered just prior to contrast administration may be effective and such a protocol could be easily used in most healthcare settings. The efficacy of this approach, however, needs validation in larger studies before it can be recommended for widespread use.

## Conclusion

Our meta-analysis suggested a significant benefit of using NaHCO_3_-based hydration for prophylaxis of CIN although the magnitude of the benefit may have been overestimated by earlier studies. However, the lack of any study to date showing superiority of saline-based hydration suggests that NaHCO_3_-based hydration should be considered the optimal hydration in high-risk patients undergoing exposure to iodinated contrast.

## Abbreviations

CI: confidence intervals; CIN: contrast-induced nephropathy; HD: hemodialysis; NAC: N-acetyl cysteine; NS: normal saline; OR: odds ratio; RCT: randomized controlled trial; SE: standard error

## Competing interests

The authors declare that they have no competing interests.

## Authors' contributions

PM was responsible for the conception and design, acquisition of data, analysis and interpretation of data, and drafting of the manuscript. DTK and AT revised the manuscript critically for important intellectual content. UT was responsible for the analysis and interpretation of data. HSG was responsible for the conception and design, acquisition, analysis and interpretation of data, and revising the manuscript critically for important intellectual content. All authors approved the final version of the manuscript and had full access to all of the data in the study.

## Pre-publication history

The pre-publication history for this paper can be accessed here:



## Supplementary Material

Additional file 1 **Table S1**. Summary of the characteristics of the 17 trials included in the meta-analysis. Click here for file
