# Supplementary material for: Sodium bicarbonate-based hydration prevents contrast-induced nephropathy: a meta-analysis
Source: BMC Med. 2009 May 13;7:23. doi: 10.1186/1741-7015-7-23 (PMC2693108; doi:10.1186/1741-7015-7-23)
Supplement: Additional file 1 — Table S1. Summary of the characteristics of the 17 trials included in the meta-analysis. [file 1741-7015-7-23-S1.doc]

Table 1. Baseline characteristics of patients enrolled in the trials included in the meta-analysis

|  | Merten^20^ | Hengel^17^ | REMEDIA^15^ | RENO^22^ | Masuda^24^ | Ozcan^25^ | Shaikh^26^ | Brar^27^ | Maioli^9^ |
| --- | --- | --- | --- | --- | --- | --- | --- | --- | --- |
| **No. of patients total** | 119 | 72 | 219 | 111 | 59 | 176 | 320 | 353 | 502 |
| Setting | Elective | Mixed | Elective | Emergency | Emergency | Elective | Elective | Elective | Elective |
| Male (%) | 75 | NA | 84 | 70 | 62 | 74 | 57 | 63 | 59 |
| Age (years) | 67.9 | NA | 70.5 | 64.5 | 75.5 | 69 | 71 | 71* | 74 |
| Diabetes (%) | 48.0 | NA | 52.0 | 23.5 | 30.9 | 44.85 | 47.2 | 44.4 | 24 |
| Baseline creatinine (mg/dL) † | 1.80 | 1.75 | 1.99 | 1.0 | 1.31 | 1.38 | 1.84 | 1.49 | 1.21 |
| eGFR (ml/min/1.73m^2^) | 43.0 | NA | 33.5 | 74.5‡ | 39.5‡ | NA | 46.8‡ | 48.1 | 42.5§ |
| Mean contrast volume used (mL) | 132 | 151.3 | 174 | 284 | 115 | 105 | 119 | 132* | 165* |
| Type of contrast used | Iopamidol | NA | Iodixanol | Iomeprol | Iopamidol | Ioxaglate | NA | Ioxilan | Iodixanol |
| **Intravenous fluid infusion protocols** |  |  |  |  |  |  |  |  |  |
| NaHCO3 total | 9 | 9 | 9 | 23 | 9 | 12 | 9 | 9 | 9 |
| NS total (ml/kg) | 9 | 9 | 24 | 12 | 9 | 12 | 9 | 9 | 24 |
| NS prior to contrast (ml/kg) | 3 | 3 | 12 | 0 | 3 | 6 | 3 | 3 | 12 |
| NAC use (%) | 0 | NA | 100 | 100 | 0 | 100 | 50 | 46 | 100 |
| Cardiac catheterization (%) | 82 | 100 | 68 | 100 | 100 | 100 | Unknown | 100 | 100 |
| Definition of CIN | Creat↑ >25% | Creat↑ >25% | Creat↑ >25% | Creat↑ >0.5mg/dl | Creat↑ >0.5mg/dl or >25% | Creat↑ >0.5mg/dl or >25%. | Creat↑ >0.5mg/dl or >25% | GFR ↓ >25% | Creat↑ >0.5mg/dl |
| CIN definition time frame (days) | 2 | 3 | 2 | 3 | 2 | 2 | 2 | 4 | 5 |

CIN: contrast-induced nephropathy. eGFR: estimated glomerular filtration rate. NA: not available; NAC: N-acetylcysteine. NaHCO3: sodium bicarbonate. NS: normal saline. * Median values. † To convert values to micromoles per liter, multiply by 88. ‡ unit = ml/min. § creatinine clearance (ml/min)

Table 1 (continued) Baseline characteristics of patients enrolled in the trials included in the meta-analysis

|  | Kim^18^ | Lin^19^ | Chen^23^ | Heguilen^16^ | Saidin^21^ | REINFORCE ^8^ | Malpica^10^ | Tamura ^11^ |
| --- | --- | --- | --- | --- | --- | --- | --- | --- |
| **No. of patients total** | 100 | 60 | 105 | 18 | 57 | 145 | 103 | 144 |
| **Setting** | Elective | Elective | NA | Elective | NA | Elective | NA | Elective |
| Male (%) | NA | 50 | NA | 63.0 | 78.9 | 78 | NA | 90 |
| Age (years) | NA | 47.7 | 70.5 | 67 | 61.6 | 71.4 | NA | 72.8 |
| Diabetes (%) | NA | 26.3 | 36.2 | NA | NA | 32.4 | NA | 58.3 |
| Baseline creatinine (mg/dL) † | 1.11 | 0.89 | NA | NA | NA | 1.49 | NA | 1.37 |
| eGFR (ml/min/1.73m^2^) | NA | NA | 52.11 | NA | NA | <60 | NA | 39.1 |
| Mean contrast volume used (mL) | NA | NA | NA | NA | NA | 139 | NA | 85 |
| Type of contrast used | Iodixanol | Iopamidol | Iohexol | Ioversol | NA | Iodixanol | NA | Iohexil |
| **Intravenous fluid infusion protocols** |  |  |  |  |  |  |  |  |
| NaHCO3 volume total | 24 | 9 | 18 | 9 | NA | 10 | NA | Single shot |
| NS total (ml/kg) | 24 | 9 | 18 | 9 | NA | 10 | NA | 24 |
| NS prior to contrast (ml/kg) | 12 | 3 | 12 | 3 | NA | 4 | NA | 12 |
| NAC use (%) | 50 | 100 | NA | 100 | 100 | NA | NA | 0 |
| Cardiac catheterization (%) | NA | 8 | NA | 100 | 100 | 100 | NA | 100 |
| Definition of CIN | Creat↑ >25% | Creat↑ >25%. | Creat↑ >0.5mg/dl or >25% | Creat↑ >25% | Creat↑ >25% | Creat↑ >0.5mg/dl or >25% | NA | Creat↑ >0.5mg/dl or >25% |
| CIN definition time frame (days) | 2 | 3 | 3 | 3 | 3 | 2 | NA | 3 |

CIN: contrast-induced nephropathy. eGFR: estimated glomerular filtration rate. NA: not available; NAC: N-acetylcysteine. NaHCO3: sodium bicarbonate. NS: normal saline. † To convert values to micromoles per liter, multiply by 88.
